# Porosity evolution at the brittle-ductile transition in the continental crust: Implications for deep hydro-geothermal circulation

**DOI:** 10.1038/s41598-017-08108-5

**Published:** 2017-08-09

**Authors:** M. Violay, M. J. Heap, M. Acosta, C. Madonna

**Affiliations:** 1 EPFL, ENAC, LEMR, Station 18, CH-1015 Lausanne, Switzerland; 2 Géophysique Expérimentale, Institut de Physique du Globe de Strasbourg, Univerisité de Strasbourg/EOST, CNRS UMR7516 Strasbourg, France; 3 Geological Institute, ETH Zurich, Sonneggstrasse, CH-8092 Zurich, Switzerland

## Abstract

Recently, projects have been proposed to engineer deep geothermal reservoirs in the ductile crust. To examine their feasibility, we performed high-temperature (up to 1000 °C), high-pressure (130 MPa) triaxial experiments on granite (initially-intact and shock-cooled samples) in which we measured the evolution of porosity during deformation. Mechanical data and post-mortem microstuctural characterisation (X-ray computed tomography and scanning electron microscopy) indicate that (1) the failure mode was brittle up to 900 °C (shear fracture formation) but ductile at 1000 °C (no strain localisation); (2) only deformation up to 800 °C was dilatant; (3) deformation at 900 °C was brittle but associated with net compaction due to an increase in the efficiency of crystal plastic processes; (4) ductile deformation at 1000 °C was compactant; (5) thermally-shocking the granite did not influence strength or failure mode. Our data show that, while brittle behaviour increases porosity, porosity loss is associated with both ductile behaviour and transitional behaviour as the failure mode evolves from brittle to ductile. Extrapolating our data to geological strain rates suggests that the brittle-ductile transition occurs at a temperature of 400 ± 100 °C, and is associated with the limit of fluid circulation in the deep continental crust.

## Introduction

The energy potential of high-enthalpy geothermal resources serves as a catalyst to probe the viability of geothermal reservoirs located in, or adjacent to, the ductile crust^1^. Feasability studies are well underway in Iceland (the Iceland Deep Drilling Project^2^), and deep geothermal projects have been proposed in Japan (Japan Beyond-Brittle Project^3^) and New Zealand (Taupō Volcanic Zone-Deep Geothermal Drilling Project^4^). It is thought that these geothermal resources—situated close to the brittle-ductile transition (BDT)—can be enhanced through hydraulic and thermal stimulation (i.e., cold fluid injections into ductile hot rocks). Such deep reservoirs have several advantages over conventional reservoirs, including (1) a very simple design, (2) the possibility to extract supercritical fluids (that have a higher power output than conventional wells for a given volumetric flow rate of steam), and (3) lower probability of induced earthquakes.

One of the main concerns for deep geothermal projects is that it has long been argued that the BDT is associated with a significant decrease in permeability in the crust^1, 5–9^. Indeed, rare *in-situ* permeability measurements^10, 11^ and numerical models^7^ suggest permeability values lower than 10^−18^ m^2^ for the ductile continental crust. By contrast, faults and fractures—efficient pathways for fluids—control the permeability of the brittle crust, the effective permeabilty of which can vary between 10^−12^ and 10^−17^ m^2^ ^7, 12, 13^. A detailed assessment of the porosity and permeability evolution across the BDT for the continential crust is therefore paramount to assess the economic potential of these deep geothermal reservoirs.

Granite is considered a major constituent of the continental crust^14^ and, as a result, there is a wealth of existing experimental data on the mechanical and hydraulical behaviour of granite. Diltancy and brittle behaviour has been well-studied^15–18^, as has ductile behaviour and the BDT^19–21^. The permeability of granite is similarly well-studied and a number of studies exist that show that microcracks and macroscopic shear and tensile fractures (i.e., brittle deformation) serve to increase permeability when measured at room temperature^22–28^. Such permeability enhancement can be reduced over time at high temperature in the presence of aqueous fluids due to crack healing and sealing^6, 29, 30^.

Studies measuring the evolution of porosity and permeability in granite as a function of strain during triaxial deformation experiments have, so far, been restricted to room temperature and brittle conditions^23, 26, 28^. However, an assessment the viability of geothermal reservoirs located in, or adjacent to, the ductile continental crust, requires measurements under the *in-situ* reservoir conditions (i.e., high-pressure and high-temperature). Here therefore we report on the mechanical behaviour and *in-situ* porosity evolution of initially-intact and thermally-shocked samples of Westerly granite at the high-temperature (600 to 1000 °C) and high-pressure (effective confining pressure of 100 MPa) conditions typical of deep (depth of ~4 km) geothermal reservoirs. The results of our study will improve our ability to analyse heat transfer between magmatic intrusions and high-enthalpy hydro-geothermal systems, to model the migration of fluids through nominally ductile crust beneath volcanoes and, more generally, will aid in understanding the nature of permeability as a function of depth in the continental crust.

## Methods

### Sample selection and preparation

**Figure 1:**
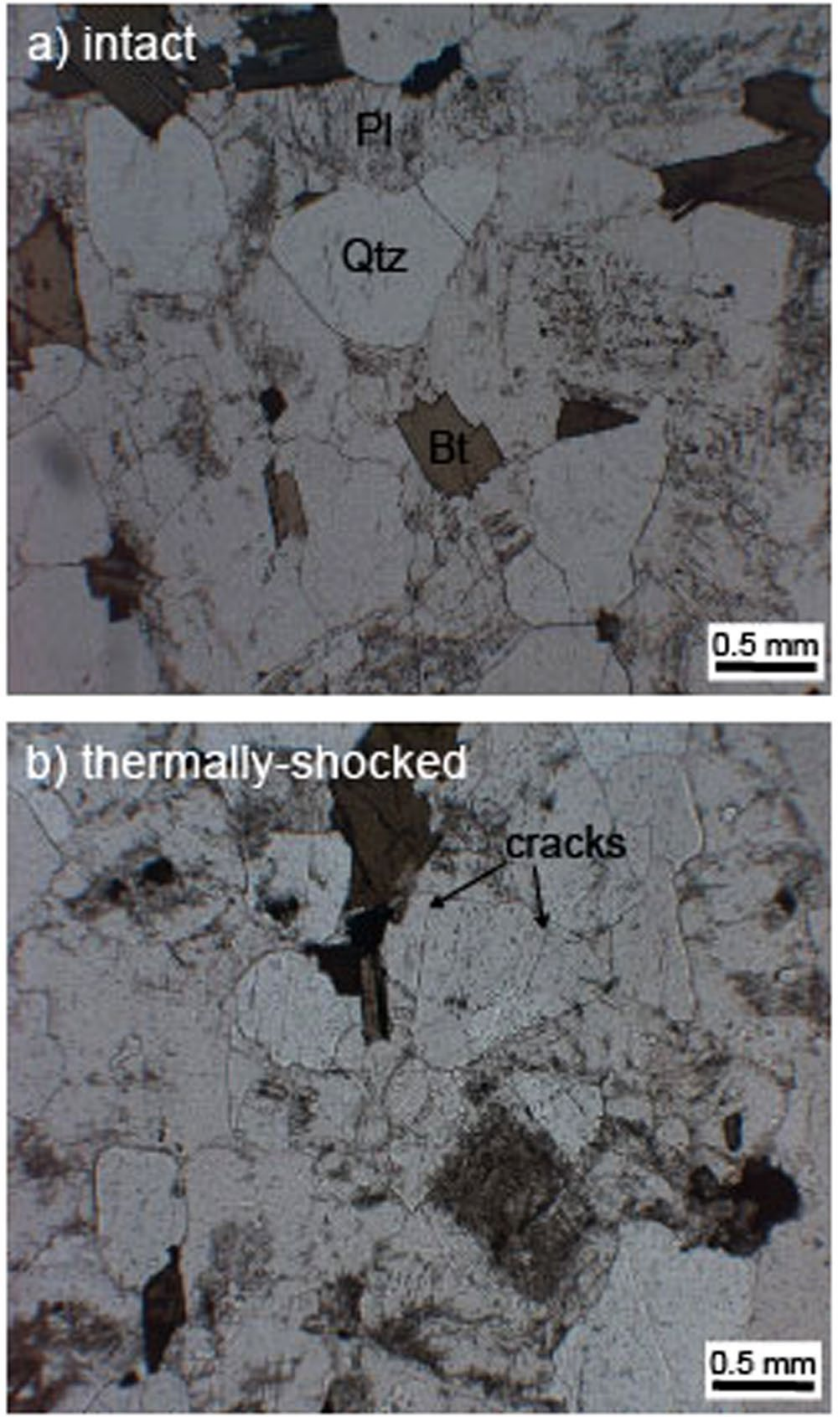
Thin section photomicrographs (taken using an optical microscope under transmitted, polarised light) of (**a**) intact Westerly granite and (**b**) Westerly granite thermally-shocked to 450 °C. Qtz – quartz; Pl – plagioclase; Bt – biotite.

The rock type used in this study—Westerly granite (USA)—was selected due to its prominance in the rock deformation literature, a consequence of its simple composition (30% quartz, 30% oligoclase, 30% microcline, and 10% biotite), low-porosity (~1.4%) (measured using a Micrometerics helium pynometer), small crystal diameter (<1 mm), and its low degree of alteration (Fig. 1a). Since Westerly granite typically has a diffuse foliation, the samples prepared for this study, 10 mm-diameter cylindrical cores that were precision-ground to a nominal length of 20 mm, were all cored in the same direction.

To better understand the influence of thermal stimulations on mechanical behaviour and porosity evolution, we thermally stressed half of our granite samples to 450 °C (i.e., below the α-β transition of quartz at room pressure, ~573 °C^31^) prior to deformation. These samples were first heated in a furnace (Carbolite Gero Ltd) at a rate of 5 °C/min up to the target temperature. The samples were then kept at 450 °C for at least 2 h, after which they were quenched in water at room temperature. This shock cooling method has been previously employed to create a thermal fracture network in laboratory samples in a geothermal context^32^. Thin section observations of the thermally stressed samples suggest that the microcracks were randomly oriented within the samples (Fig. 1b). Sample porosity following thermal stressing was ~2.7% (i.e., twice the value of the unheated samples). All samples were then dried for 48 h at 70 °C prior to deformation.

### Experimental methods

The granite samples were placed between alumina and zirconia pistons and jacketed with either copper or iron, depending on the target experimental temperature. Experimental temperatures ranged between 600 and 1000 °C. Although these temperatures are higher than those expected in a geothermal reservoir, it is commonplace to use higher temperatures in laboratory deformation experiments to compensate for the discrepancy between laboratory and natural strain rates. To minimise the jacket contribution on the force measurement, copper was used at temperature 600 and 700 °C, whereas iron jackets were used at temperatures between 800 and 1000 °C. Samples were then deformed in a triaxial servo-controlled, gas-medium apparatus from Paterson instruments^33^. The confining pressure (P_c_) and pore fluid (argon) pressure (P_f_) were 130 and 30 MPa, respectively, corresponding to an effective confining pressure (P_c_^eff^) of 100 MPa (assuming a simple effective pressure law). The chosen experimental pressure, which corresponds to a depth of ~4 km, is therefore representative for deep geothermal reservoirs^3, 4^. Once the target pressure and temperature were imposed, the sample was deformed in compression at an axial strain rate of 1 × 10^−5^ s^−1^ up to an axial strain of ~7%. Axial force and axial strain were measured using an internal load cell and a linear variable differential transducer (LVDT), respectively. Force, converted to stress using the sample radius, was corrected from the contribution of jacket and from the change in sample radius during deformation (following the method described in Violay et al.^8^). Creep equations for copper and steel, determined by Frost and Ashby^34^, were used to calculate the contributions of the jacket to the data, which were subsequently subtracted. Measurements of axial displacement were converted to axial strain using the sample length; displacement measurements were corrected for apparatus distortion. Differential stress and axial strain were measured with an accuracy of 2 MPa and 0.1%, respectively.

Porosity change during deformation was measured by means of a pore fluid volumometer. The method consisted of monitoring the pore fluid volume variation while keeping the pore pressure constant^35^. The volume resolution was 0.5 mm^3^. The pore fluid volume (i.e., porosity) was corrected for (1) any leaks, (2) apparatus distortion (i.e., pore volume changes due to deformation of the pore fluid system during loading, correction of 0.03 mm^3^/mm), and (3) the temperature gradient between the volumometer and the sample (for details see Fischer and Paterson^5^). Noise due to the vibration of the volumometer piston was filtered using the 1D filter function in MATLAB.

Sample drainage is an important consideration in the deformation of low-porosity rocks. Although intact granite has a permeability as low as ~10^−23^–10^−19^ m^2^ ^5, 24, 27^, the presence of microcracks can increase permeability by up to seven orders of magnitude^27^. All our experimental temperatures are all above the α-β transition of quartz at the experimental pressure (~600 °C); the samples will therefore contain a pervasive microcrack network due to the thermal expansion as α-quartz transforms to β-quartz^31^. Of interest here is whether such microcracks can remain open at the high effective pressure (P_c_^eff^ = 100 MPa) used in this study, and therefore provide the permeability required for drained deformation. To this end, we measured the permeability of our samples at the experimental pressure (P_c_^eff^ = 100 MPa) and a range of temperatures (from 500 to 900 °C) using the pulse-decay method^22^ (see Supplmentary Materials for more details). Permeability was measured to be ~1 × 10^−19^ and ~4 × 10^−19^ m^2^ at 600 and 900 °C, respectively. The fourfold increase in permeability between 600 and 900 °C is interpreted here as a result of an increase in thermally-induced microcracks (see Supplmentary Materials). Importantly, these values of permeability are higher than those measured for intact Westerly granite^22^ under the same pressure (P_c_^eff^ = 100 MPa): 2–4 × 10^−20^ m^2^, implying that some thermally-induced microcracks remain open at high pressure. If the minimum timescale for argon diffusion through the sample can be approximated using *t* = *l* ^2^ × *η* × *β/k* [Fischer and Paterson^5^], the time required for an argon atom to travel half the length of the sample is 500 s, assuming a length *l* of 0.02 m, a dynamic viscosity of argon *η* of 10^−4^ Pa s, a storage capacity per unit volume *β* of 10^−8^ Pa^−1^, and an average permeability of 2 × 10^−19^ m^2^. This timescale is considerably shorter than the total duration of an experiment (5000 to 7000 s). We consider our samples as drained during our deformation experiments.

Following deformation, the samples were unloaded and the pressure and temperature were reduced to ambient conditions. To assess sample failure mode, the deformed samples were scanned using X-ray computed tomography (CT). The CT scans were performed employing a phoenix v|tome|x s 240 X-ray scanner (GE Sensing & Inspection Technologies GmbH, Wunstorf, Germany) at ETH Zurich, Switzerland. Scanning resolution was 27.50 µm voxel edge length. The current and voltage used were 190 µA and 140 KV, respectively. Exposure time per image was 250 ms. The volume reconstruction was performed using the software datos|x (GE Sensing & Inspection Technologies GmbH, Wunstorf, Germany) at ETH Zurich, Switzerland. The original reconstructed images (32 bit float) were downscaled to unsigned 16-bit format for further data processing and generation of the tiff-stack.

To better understand the operative microstructural deformation mechanisms, we also prepared 30 μm-thick polished thin sections of each sample (if deformation was localised, the samples were cut perpendicular to the shear plane). The thin sections were examined using an optical microscope (under transmitted, polarised light) and a backscattered scanning electron microscope (SEM; XLF-30-FEG from Phillips; acceleration voltage was 12 kV) at EPFL, Switzerland.

## Results

### Mechanical data

#### Initially-intact mechanical data

**Figure 2:**
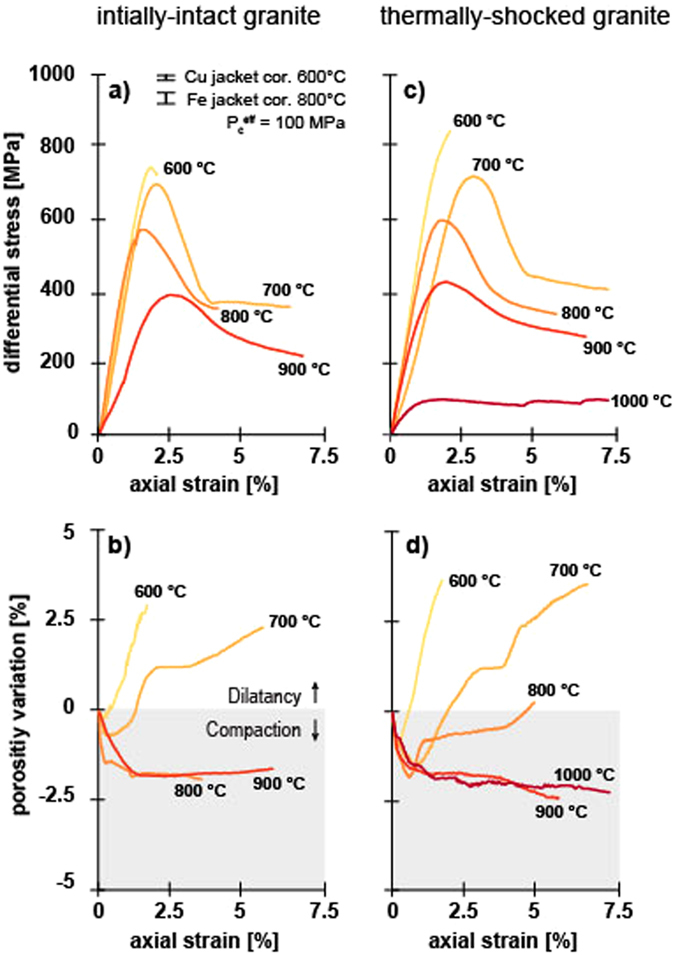
Mechanical data and porosity evolution for triaxial deformation experiments performed at temperatures between 600 and 1000 °C and an effective confining pressure of 100 MPa. Samples were deformed in compression at an axial strain rate of 10^−5^ s^−1^. (**a**) and (**b**) show the mechanical behaviour and porosity evolution, respectively, for samples of initially-intact Westerly granite. (**c**) and (**d**) show the mechanical behaviour and porosity evolution, respectively, for samples of Westerly granite thermally-shocked to 450 °C. Porosity data were smoothed with the 1D digital filter of MATLAB.

The stress-strain curves for the initially-intact samples (so called because they now contain a thermal microcrack network as a result of exposure to the experimental temperature) are shown in Fig. 2a. In all experiments, the differential stress first increased linearly with increasing strain (i.e., elastic). Following elastic deformation, the differential stress was a non-linearly decreasing function of strain (i.e., strain hardening) prior to a peak stress (Fig. 2a). The samples entered a strain softening phase (i.e., stress drop) following the peak stress (Fig. 2a). The mechanical behaviour of all four samples is indicative of a brittle failure mode. Peak stress was reduced from 815 to 719 MPa as temperature was increased from 600 and 900 °C, respectively (Fig. 2a and Table 1). We also note that the magnitude of the stress drop following the peak stress was reduced as temperature was increased (Fig. 2a).

**Table 1:** Summary of experimental conditions and results. TS – sample thermally-shocked to 450 °C; I – initially-intact sample; D – dilation; C – compaction.

Sample number	Type	Experimental temperature [°C]	Confining pressure, Pc [MPa]	Pore fluid Pressure, Pf [MPa]	Peak Differential stress at failure [MPa]	Failure mode	Porosity evolution
G1	TS	700	130	30	769	Brittle	D
G2	TS	800	130	30	609	Brittle	D
G3	TS	600	130	30	854	Brittle	D
G4	TS	900	130	30	436	Brittle (transitional)	C
G5	TS	1000	130	30	Ductile	Ductile	C
G10	I	700	130	30	716	Brittle	D
G12	I	800	130	30	568	Brittle	C
G13	I	900	130	30	384	Brittle (transitional)	C
G15	I	600	130	30	815	Brittle	D

The porosity change curves as a function of axial strain for these experiments are shown in Fig. 2b. We find that the porosity of the samples deformed at 600 and 700 °C first decreased (up to an axial strain of ~0.5%), due to the closure of microcracks, before increasing due to microcrack nucleation, growth, and coalescence (Fig. 2b). The porosity of the samples deformed at 800 and 900 °C both decreased by ~2% up to an axial strain of ~1%, before remaining pseudo-constant for the remainder of the experiment (Fig. 2b). At the end of the experiment, the samples deformed at 600 and 700 °C had increased, while the samples deformed at 800 and 900 °C had decreased (Fig. 2b).

#### Thermally-shocked mechanical data

The stress-strain curves for the thermally-shocked samples are shown in Fig. 2c, and are qualitatively similar to the corresponding experiments (i.e., temperatures between 600 and 900 °C) on the initially-intact samples (Fig. 2a). We note that the experiment at 600 °C was arrested prior to the peak stress because the maximum load of the Paterson was almost reached (maximum load 100 kN, corresponding to a stress of 1 GPa on a 10 mm-diameter sample). As for the initially-intact samples, the peak stress and the magnitude of the stress drop following the peak stress were reduced as temperature was increased (Fig. 2c and Table 1). However, the mechanical behaviour of the sample deformed at 1000 °C was markedly different to those deformed at temperatures between 600 and 900 °C (Fig. 2c). The maximum stress reached during the experiment was considerably lower and the strain softening following the peak stress was considerably reduced (Fig. 2c).

The porosity change curves as a function of axial strain for these experiments are shown in Fig. 2d. The porosity of the samples deformed at temperatures between 600 and 800 °C first decreased before increasing up to the maximum axial strain imposed on the sample (Fig. 2d). The initial decrease in porosity due to microcrack closure is higher for the thermally-shocked samples (Fig. 2d) than for the initially-intact samples (Fig. 2b). The porosity of the samples deformed at 900 and 1000 °C both decreased by ~2% up to an axial strain of ~1%, before remaining pseudo-constant for the remainder of the experiment (Fig. 2d). At the end of the experiment, the samples deformed at 600 to 800 °C had increased, while the samples deformed at 900 and 1000 °C had decreased (Fig. 2d).

### X-ray computed tomography (CT)

**Figure 3:**
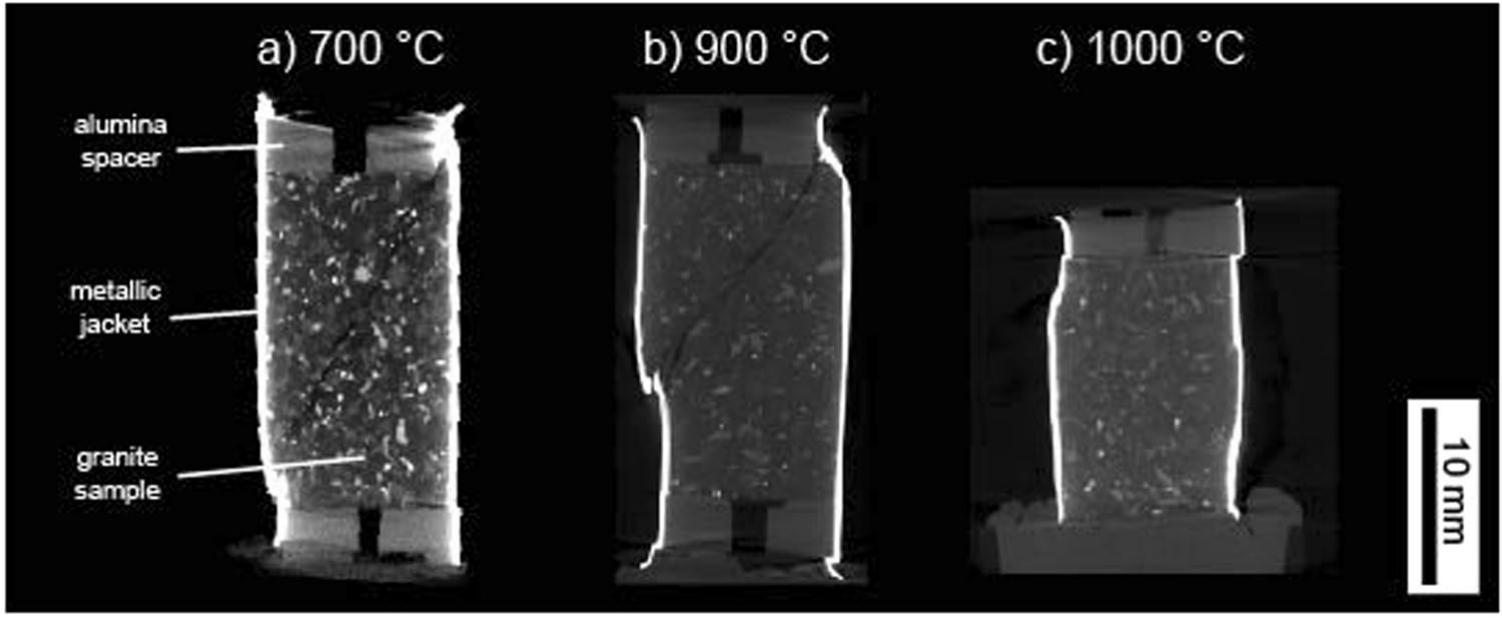
X-ray computed tomographic images of the samples of Westerly granite (thermally-shocked to 450 °C) deformed at an effective confining pressure of 100 MPa and an axial strain rate of 10^−5^ s^−1^ (the deformation experiments shown in Fig. 2). (**a**) Sample deformed at 700 °C. (**b**) Sample deformed at 900 °C. (**c**) Sample deformed at 1000 °C.

To investigate rock failure mode, we performed CT on our deformed samples. We consider here that a brittle mode of deformation is confirmed by the presence of strain localisation (i.e., shear fractures) and that ductile deformation is confirmed by the absence of strain localisation^36^. We find that the samples (intact and thermally-shocked) deformed at temperatures up to 900 °C contain throughgoing shear fractures (i.e., brittle) (see examples in Fig. 3a and b). The shear bands are inclined at 30 to 45° to the maximum principal stress direction in all of the brittle samples (Fig. 3a and b). However, the sample deformed at 1000 °C contains no evidence of strain localisation at the sample lengthscale (i.e., ductile; Fig. 3c).

### Microstructural observations

**Figure 4:**
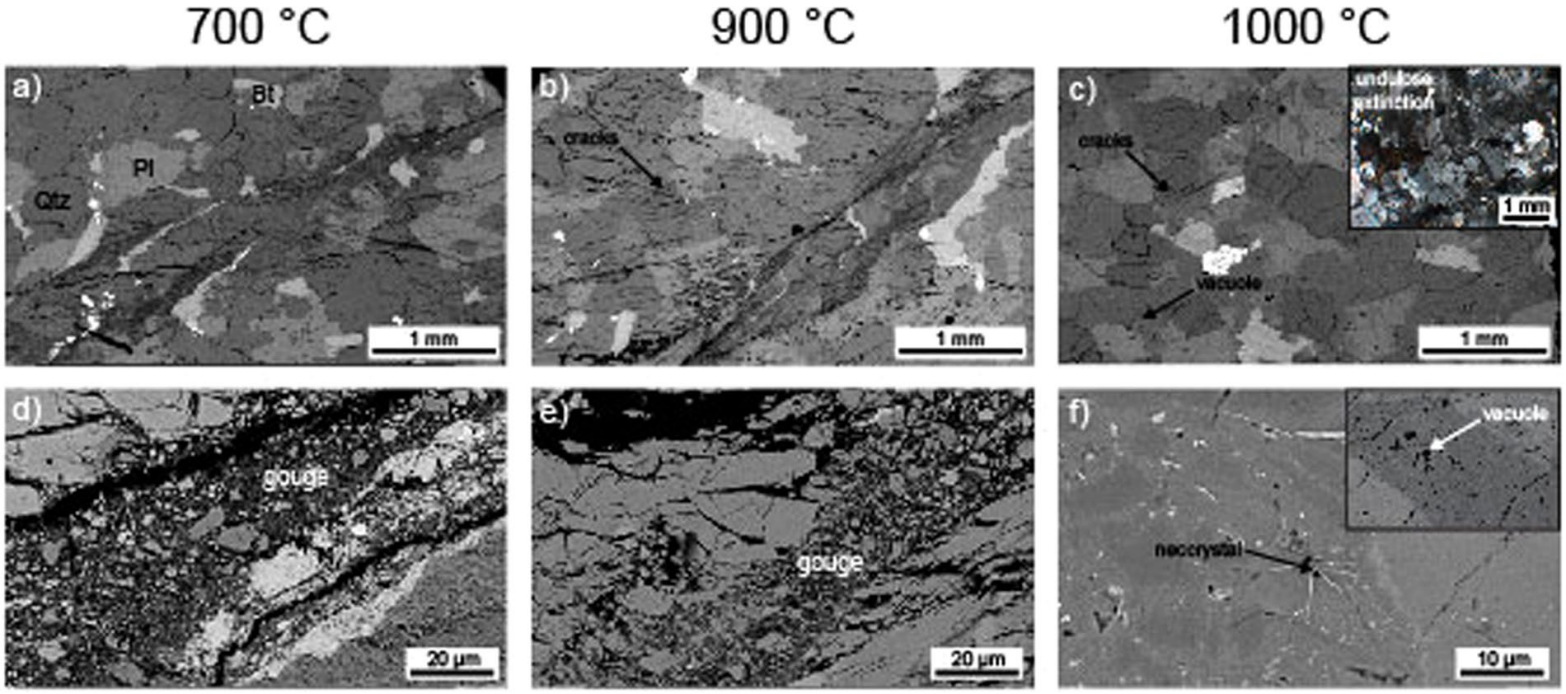
Backscattered scanning electron photomicrographs (SEM) of the samples of Westerly granite (thermally-shocked to 450 °C) deformed at an effective confining pressure of 100 MPa and an axial strain rate of 10^−5^ s^−1^ (the deformation experiments shown in Fig. 2). (**a**) and (**d**) are SEM images of the sample deformed at 700 °C, showing a shear band containing fine-grained gouge. Qtz – quartz; Pl – plagioclase; Bt – biotite. (**b**) and (**e**) are SEM images of the sample deformed at 900 °C, showing a shear band containing fine-grained gouge. (**c**) and (**f**) are SEM images of the sample deformed at 1000 °C, for which no strain localisation was found. Inset in panel (c) is an optical microscopic image showing undulose extinction in crystals of quartz. Panel (f) shows a neocrystal; the inset shows vacuoles within a feldspar crystal.

To better understand the operative microstructural deformation mechanisms, we examined thin sections of our samples under an optical microscope and an SEM. We find that the samples (intact and thermally-shocked) deformed at temperatures between 600 and 900 °C contain wide (0.5 to 1 mm-thick) anastomosing shear bands containing fine-grained (0.1 to 100 μm) gouge (Fig. 4a,b,d and e). The crystals adjacent to the shear band are typically densely fractured (Fig. 4a and b). We also find that biotite crystals and high-density oxides are elongated parallel to the shear direction within the shear bands (Fig. 4a,b,d and e).

No obvious shear fracture was observed in the sample deformed at 1000 °C (Fig. 4c). Sample deformation was accomodated by a combination of microcracking and plastic deformation on the microscale (Fig. 4c). In particular, we see many microcracks within quartz crystals (Fig. 4c). Biotite crystals show evidence of kinking, twinning, and thermal decomposition (vacuoles; Fig. 4e and f), and quartz crystals show evidence of strong undulose extinction and rare deformation lamellae (see inset in Fig. 4c). We also note the presence of incipient recrystallization of feldspar (Fig. 4f). Additional microstructural images of the sample deformed at 1000 °C are provided in the Supplementary Materials.

## Discussion

### Influence of temperature on the strength of granite

Our high-pressure, high-temperature triaxial deformation experiments show that the strength of Westerly granite is reduced as temperature is increased (Fig. 2), as previously observed in experiments on Westerly granite^20, 21^. For example, peak stress is reduced from 815 to 719 MPa as temperature is increased from 600 and 900 °C, respectively (Fig. 2a and Table 1). We interpret the observed reduction in strength as the result of the increased efficiency in crystal-plastic processes^20^ (Fig. 4) and stress corrosion cracking^37, 38^ at high temperature.

### Influence of thermally-shocking samples prior to high-pressure, high-temperature deformation

The uniaxial compressive strength^39, 40^, elastic moduli^41^, compressibility^42^, elastic wave velocities^43^, and permeability^25, 28, 44^ of granite are strongly influenced by the presence microcracks. However, the strength of granite thermally-stressed to 700 °C and deformed at room temperature under triaxial conditions (for Pc > 50 MPa) was found to be similar to intact granite^28^. Similar conclusions were drawn by Wong^21^.

Our high-pressure, high-temperature triaxial deformation experiments show that there is no measurable difference in peak stress between the samples thermally-shocked to 450 °C prior to deformation and the initially-intact samples (Fig. 2 and Table 1), in accordance with the studies of Wong^21^ and Wang *et al*.^28^. We also find that thermally-shocking samples to 450 °C prior to deformation did not change the failure mode and operative micromechanisms of deformation (Figs 3 and 4).

Although microcracks can remain open at high-pressure, as evidenced by permeability data for thermally microcracked rocks^28, 45^ (see also the data presented in the Supplemetary Materials), the longest, most-deleterious microcracks—those most likely to influence rock strength—may readily close at high confining pressures. Therefore, although the samples thermally-shocked prior to deformation contain more microcracks than the initially-intact samples, these microcracks are likely small and do not greatly influence rock strength as a result^28^. We also highlight that the minimum experimental temperature, 600 °C, is higher than the thermal stressing temperature (450 °C). Another possibility for the observed similarity between the initially-intact and the thermally-shocked samples is therefore that the microcrack densities for both types of sample were similar at each experimental temperature.

### Porosity evolution during high-pressure, high-temperature deformation of granite: Implications for permeability

The transition from a brittle to a ductile mode of deformation strongly influences the evolution of porosity and permeability as a function of inelastic strain^8, 9, 46, 47^. However, and as outlined above, experimental evidence for porosity evolution during the high-pressure, high-temperature deformation of granite is scarce, although data exist for basalt^9^ and andesite^48^. Our experiments show that granite is brittle between temperatures of 600 and 900 °C at an effective confining pressure of 100 MPa (Fig. 2). Indeed, localised deformation—the hallmark of a brittle failure mode^36^—is observed in each sample following deformation (Figs 3 and 4). However, despite the staunchly brittle failure mode between 600 and 900 °C, the evolution of porosity during deformation was markedly different at 900 °C (Fig. 2). At 600 and 800 °C, porosity first decreased, due to the closure of microcracks, followed by a porosity increase up to the maximum strain imposed on the samples (Fig. 2). This porosity increase is related to the nucleation, growth, and coalesence of microcracks, as observed in many other studies on the brittle deformation of low-porosity rocks such as granite^15, 16, 23, 26, 28^.

However, the porosity of the samples deformed at 900 °C (and the initially-intact sample deformed at 800 °C) remained pseudo-constant following the initial decrease in porosity due to microcrack closure (Fig. 2). In these samples, the porosity increase due to microcracking (that led to the formation of a shear fracture; Figs 3 and 4) was likely counterbalanced by the porosity decrease due to crystal-plastic processes^20^ (Fig. 4). The transition from an initial decreasing porosity to a pseudo-constant porosity with increasing deformation suggests that the vast majority of the connected porosity is closed, which was presumably ~2-2.5% at the start of the experiment, at an axial strain of just ~1% (Fig. 2). Although the mechanical data (Fig. 2) and macroscopic (Fig. 3) and microstructural (Fig. 4) observations suggest a brittle failure mode, deformation at 900 °C could be considered as “transitional”.

Deformation at 1000 °C was ductile, as evidenced by the absence of strain localisation^36^ (Figs 3 and 4) (although we note that there is some post-peak strain softening, see Fig. 2). The mechanical data (Fig. 2) and microstructural observations (Fig. 4) highlight that crystal-plastic processes were more efficient at 1000 °C^20^ (Fig. 4), although we note that the porosity evolution was similar as for the samples deformed at 900 °C (Fig. 2), described above.

From these data and observations we can conclude that brittle behaviour associated with dilatancy (temperatures between 600 and 800 °C) will likely increase permeability, while brittle behaviour close to the BDT transition associated with compaction (temperatures between 800 and 900 °C) and ductile deformation at 1000 °C will likely decrease permeability. Therefore, as concluded for the basaltic oceanic crust^9^, the BDT in granite is also likely to be associated with a significant decrease in permeability in the continental crust.

### The brittle-ducile transition at crustal strain rates

With increasing depth and temperature, rocks can undergo a transition in failure mode from localised brittle deformation to distributed ductile flow. In the brittle domain, rock strength depends weakly on strain rate and temperature, but strongly on confining pressure^49^. The sensitivity of strength to strain rate and temperature are much more pronounced in the ductile domain^50^. As a result, it is necessary to extrapolate the results of our laboratory experiments—performed at a laboratory strain rate of 10^−5^ s^−1^—to geological strain rates, which are on the order of 10^−12^ to 10^−15^ s^−1^. Since such natural strain rates are impracticable in the laboratory, it is commonplace to perform experiments at higher temperatures to compensate (as was the case for the experiments of this study^50^).

Mechanical experiments conducted on granite have proposed constitutive laws to describe deformation in the brittle and ductile domain. Brittle failure by localised shear fracturing has been described using a simple Coulomb failure criterion (i.e., friction law)^18, 51, 52^. Since the potential deformation micromechanisms are numerous (e.g., microfracturing, intracristalline plasticity, diffusion/dissolution creep, and partial melting), the description of transitional (or “semi-ductile”) and ductile behaviour is correspondingly complex^20, 53–57^. Strength in the ductile (and transitional) regime has often been expressed via a constitutive temperature-dependent power law. The application of such constitutive laws to experimental data suggest that, at a strain rate of 10^−14^ s^−1^, granite may deform in the brittle field to temperature up to 400 ± 100 °C, above which ductile deformation will dominate^58, 59^. The friction law and temperature-dependent power law obtained for our experimental data (see Supplementary Materials) are in good agreement with the wealth of previously published data on fine-grained granites. We therefore contend that the insight provided by our experimental data, and in particular the porosity evolution during deformation under brittle and ductile conditions in the laboratory, are relevant to deformation in the crust under geological conditions.

### Implications for deep geothermal energy

Engineering a deep geothermal reservoir will require the creation of fracture network between the injection and the production wells^3^. Two fracturing techniques could be considered. The first method is hydrofracturing, a method commonly employed in the development of Enhanced Geothermal Systems (EGS). Another possibility is to create fractures by thermal stimulation (i.e., cold water injection)^60^. Our experiments have shown that additional thermally-induced microcracks may not significantly influence strength or failure mode under the *in-situ* conditions (Fig. 2). However, permeability may be transiently enhanced, as evidenced by the increase in porosity loss in the samples thermally-shocked to 450 °C, compared to the initially-intact samples, during the initial stages of deformation (suggesting that the porosities of the thermally-shocked samples were higher at the start of the experiments) (Fig. 2). However, although permeability-enhancing brittle behaviour is likely to occur at the pressures and depths anticipated for deep geothermal reservoirs, we note that ductile behaviour and brittle behaviour close to the BDT is associated with a porosity reduction that is likely to reduce permeability^61^. We also note that the presence of hydrothermal fluids under high-pressure, high-temperature conditions will likely reduce permeability over time due to fracture healing and sealing (we used argon as the pore fluid for our deformation experiments)^30^. The data presented herein highlight the challenge associated with maintaining porosity and permeability in reservoirs at, or close to, the continental BDT.

## Conclusions

Our study informs on porosity evolution with deformation around the brittle to ductile transition in the continental crust. We performed high-pressure (effective confining pressure of 100 MPa), high-temperature (600–1000 °C), triaxial experiments in which porosity was monitored during deformation. Mechanical data and post-mortem sample characterisation suggest that:

1. The failure mode of the granite was brittle up to a temperature of 900 °C (shear band formation) but ductile at 1000 °C (no strain localisation).
2. Only deformation up to 800 °C was dilatant.
3. Deformation at 900 °C was brittle (a shear fracture formed) but associated with net compaction due to an increase in the efficiency of crystal plastic processes.
4. Ductile deformation at 1000 °C was compactant.
5. Thermally-shocking the granite to 450 °C prior deformation did not influence the strength or failure mode of granite during high-temperature, high-pressure deformation.
6. Extrapolating our experimental data to geological strain rates yields a friction law and temperature-dependent power law in good agreement with the wealth of previously published data on fine-grained granites.
7. The BDT is likely associated with a significant decrease in permeability in the continental crust.
8. The data presented herein highlight the challenge associated with maintaining porosity and permeability in reservoirs at, or close to, the continental BDT.

### Data availability

The data of Fig. 2 can be requested from the corresponding author: marie.violay@epfl.ch.

## Electronic supplementary material

Supplementary Materials
