# Supplementary material for: Porosity evolution at the brittle-ductile transition in the continental crust: Implications for deep hydro-geothermal circulation
Source: Sci Rep. 2017 Aug 9;7:7705. doi: 10.1038/s41598-017-08108-5 (PMC5550508; doi:10.1038/s41598-017-08108-5)
Supplement: Supplementary file 1 — Supplementary Materials [file 41598_2017_8108_MOESM1_ESM.pdf]

Supplementary Materials for:
Porosity evolution at the brittle-ductile transition in the continental crust: Implications for deep hydro-geothermal circulation.

Violay M., Heap M.J., Acosta M., and Madonna C.

*To whom correspondence should be addressed. Email: marie.violay@epfl.ch

This file includes text and figures divided into four supplementary items:

1. SM1: Constitutive laws

Although not central to our study (since many dedicated studies exist), we determined constitutive laws using our data (for the ductile and brittle regimes) to provide a first-order estimate of brittle-ductile transition in the continental crust, to compare with previously published estimates (e.g., *Heard et al.* [*1968*]; *Byerlee, et al.* [1*978*]; *Jaoul et al.* [*1984*]; *Kronenberg and Tullis* [*1984*]; *Rybacki and Dresen* [*2000*]) (see Supplementary Item 2).

In the ductile regime, we analyse strength by computing effective parameters using a power law of the form:

$$\dot{\varepsilon} = A.\sigma^{n}.\exp\left(-{}^{Q}/_{RT}\right) \quad (1)$$

where $\dot{\varepsilon}$ is the strain rate, σ is the stress, *A* is a material constant, *n* is the stress exponent, *Q* is the activation energy, and *R* is the gas constant. To determine the apparent *n*, *Q*, and *A* for Equation (1), we used experimental data from our experiments performed at temperatures of 800, 900, and 1000 °C (i.e. ductile and "transitional" behaviour).

The strain rate dependence was determined from strain rate steps and stress relaxations that were applied during deformation at 1000 °C. The stress exponent *n*,

determined from the linear regression of the log (stress) versus the log (strain rate), was found to be 8.5 (Figure SM1a). This value is relatively high compared to other rock samples deformed at higher temperatures and pressures [*Evans et al*., 1990] and is the result of several operative micromechanisms – namely microcracking and intracrystalline plasticity (see Figure 4 in the manuscript). The activation energy, found to be 497 kJ/mol, was determined from a least squares fit of log (stress) versus 1/T at strain rate of 10^{-5} s^{-1} (Figure SM1b).

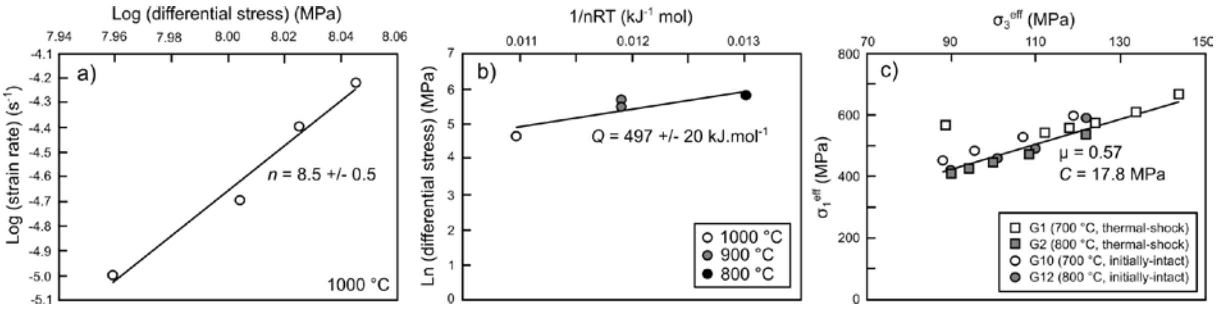

Figure SM1. A) Log strain rate as a function of log stress for the sample deformed at 1000 °C. B) Stress as a function of the reciprocal temperature at strain rate of 10^{-5} s^{-1} for the "transitional" and ductile samples. C) Maximum effective stress σ_1 as a function of the minimum effective stress σ_3 for the samples deformed in the brittle regime (data acquired by implementing pore pressure steps (see text for details). All the data in Figure SM1 are unique to this study.

In the brittle field, the friction strength and cohesive strength were determined using a simple Coulomb failure criterion at $\dot{\varepsilon}$ = 10^{-5} s^{-1}. The sensitivity of σ_3 on σ_1 was determined by implementing pore pressure steps at the end of the experiments. We only used experimental data from our experiments performed at temperatures between 600 °C and 800 °C (i.e. purely brittle behaviour and the "transitional" behaviour at 800 °C). A linear fit between σ_1^{eff} and σ_3^{eff} gives a slope K_p = 4.04 and σ_0 = 60 MPa. The fracture angle and the cohesion characteristics of these parameters are given by the following [*Violay et al.,* 2012]:

$$tan(\mu) = sin^{-1}\left(\frac{K_p - 1}{K_p + 1}\right) \quad (2)$$

$$C = \frac{\sigma_1 - \sigma_3.K_p}{2.\sqrt{K_p}} \quad (3)$$

From Equation 2 and 3, the coefficient of friction, μ = 0.57 and cohesive strength C = 17.8 MPa (Figure SM1c). These values are lower than those suggested by *Byerlee* [1978] ($\tau = 50 + 0.6.\sigma n$), but agree with previous studies on rocks at high temperature ($0.43 < \mu < 0.6$) [*Violay et al.,* 2012].

2. SM2: extrapolation to natural strain rates of 10^{-14} s^{-1}

Although not explicitly the goal of our study, we made a simplified first-order estimate of the depth of the brittle-ductile transition in the continental crust to constrain the maximum depth at which hydrothermal fluids circulate. The friction law and power law were used to calculate the strength as a function of depth, assuming a strain rate of 10^{-14} s^{-1} and a high thermal gradient of 100 °C/km (geothermal gradient in Japan, *Asanuma et al.* [*2012*]).

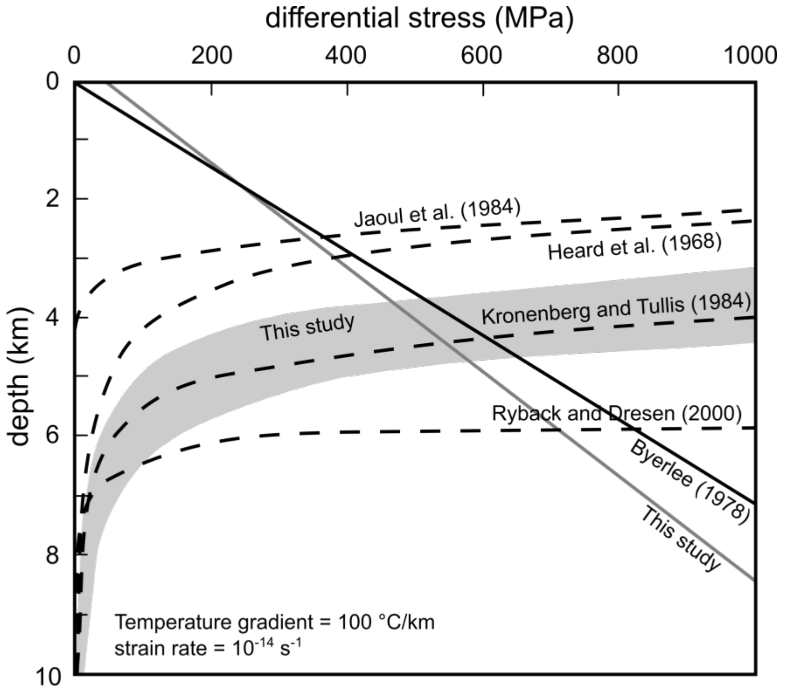

Figure SM2: Differential stress as a function of depth for a continental crust with a high geothermal gradient of 100 °C/km. A crustal strain rate of 10^{-14} s^{-1} was used in the calculations. The shaded area shows data from our study. We also provide published curves from Heard et al. [*1968*], *Byerlee, et al.* [1*978*], *Jaoul et al.* [*1984*], *Kronenberg and Tullis* [*1984*], *and Rybacki and Dresen* [*2000*].

The validity of the extrapolation strongly depends on the accuracy of the determination of *μ*, *n*, and *Q* in the constitutive relations. Extrapolation was compared to previously published constitutive models for the continental crust (experimental data on single crystal (quartz and feldspar), and multiphase rock (quartzite) [*Heard et al., 1968; Byerlee, et al., 1978; Jaoul et al., 1984; Kronenberg and Tullis, 1984; Rybacki and Dresen, 2000*]. We estimate that granite may deform in the brittle field up to 400 ± 100 °C at 10^{-14} s^{-1} (Figure SM2). If these extrapolations are correct, hydrothermal fluids might circulate, at least transiently, through the continental crust down to a depth of 3 to 5 km.

3. SM3: Microstructures of the sample deformed at 1000 °C

We provide in the following figure (Figure SM3) additional microstructural images of the sample deformed at 1000 °C.

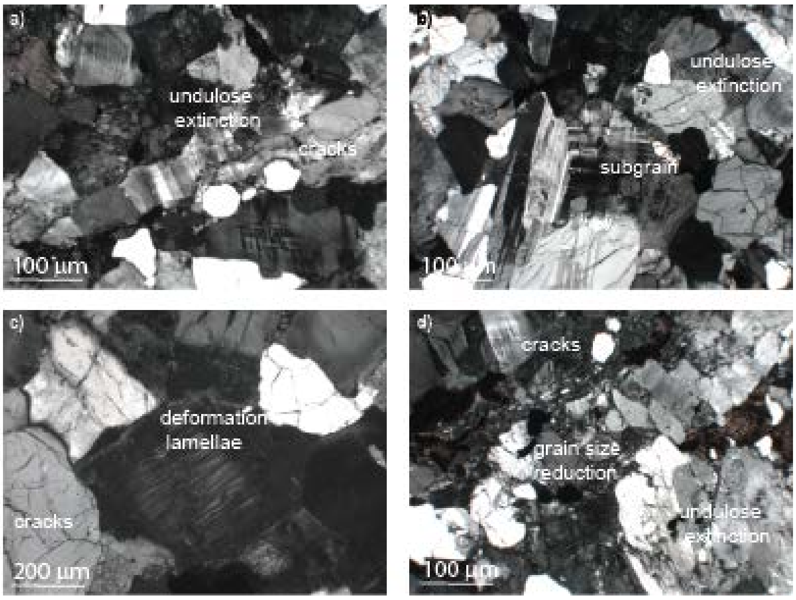

Figure SM3: Thin section photomicrographs (taken using an optical microscope under transmitted, polarised light) of the sample deformed at 1000 °C. Sample deformation was accommodated by a combination of microcracking and plastic deformation on the microscale. In particular, we see many microcracks (panels (a) and (d)) and strong undulose extinction and rare deformation lamellae (panels (a), (b), and (d)) within quartz crystals. We also show evidence of subgrains within feldspar crystals (panel (b)).

4. SM4: Permeability of Westerly granite at high pressure and high temperature

The Paterson apparatus at ETH is equipped with a pore pressure intensifier/volumometer with upstream and downstream pore-fluid connections. The volumometer can be used to determine permeability using the pulse-decay method [*Brace et al*., 1968]. Permeability is determined using this method by monitoring the equilibration of pore fluid (argon gas) pressure across a permeable sample separated by two fixed-volume reservoirs. In general, the pressure in the upstream pore fluid reservoir is first increased and its decay across the sample subsequently monitored.

Permeability measurements were performed prior to sample deformation at an effective pressure of 100 MPa (a confining pressure of 130 MPa and an equilibrium pore pressure of 30 MPa) and temperatures ranging from 500 to 900 °C. A pore pressure difference of about 1 MPa between the upstream and downstream reservoirs was applied; the decay of this pressure change with time was fitted with an inverse exponential decay in order to calculate the permeability, following the method described in *Bakker et al*. [2015].

Initial permeability increased with increasing temperature from 5×10^{-20} (at 500 °C) to 3.1×10^{-19} m^2 (at 900 °C) (Figure SM4). We interpret the increase in permeability with increasing temperature as a consequence of an increase in thermally induced microcracks. Values of permeability for the samples measured at temperatures higher than the quartz α-β transition [*Glover et al*., 1995] are higher than those measured for intact Westerly granite under the same pressure (P_c^{eff} = 100 MPa): 2-4 $\times 10^{-20}$ m^2 [*Brace et al*., 1968], implying that some thermally-induced microcracks remain open at high pressure.

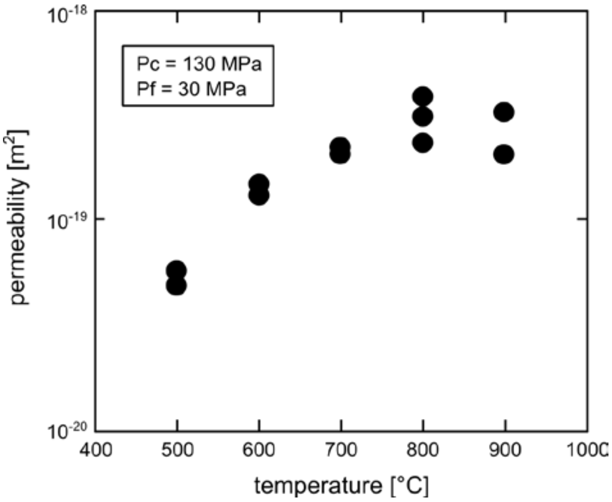

Figure SM4: Permeability evolution in Westerly granite at an effective pressure of 100 MPa as a function of temperature.

References:

Bakker, R. R., Violay, M. E., Benson, P. M., & Vinciguerra, S. C. (2015). Ductile flow in sub-volcanic carbonate basement as the main control for edifice stability: New experimental insights. Earth and Planetary Science Letters, 430, 533-541.

Brace, W., Walsh, J. B., & Frangos, W. T. (1968). Permeability of granite under high pressure. Journal of Geophysical research, 73(6), 2225-2236.Byerlee, J. (1978). Friction of rocks. In Rock friction and earthquake prediction (pp. 615-626). Birkhäuser Basel.

Evans, B., Fredrich, J. T., & Wong, T. F. (1990). The brittle-ductile transition in rocks: Recent experimental and theoretical progress. The brittle-ductile transition in rocks, 1-20.

Glover, P.W.J., Baud, P., Darot, M., Meredith, P.G., Boon, S.A., LeRavalec, M., Zoussi, S., & Reuschlé, T. (1995). α/β phase transition in quartz monitored using acoustic emissions. *Geophysical Journal International*, *120*(3), 775-782.

Heard, H. C., & Carter, N. L. (1968). Experimentally induced'natural'intragranular flow in quartz and quartzite. *American Journal of Science*, *266*(1), 1-42.

Jaoul, O., Tullis, J., & Kronenberg, A. (1984). The effect of varying water contents on the creep behavior of Heavitree quartzite. *Journal of Geophysical Research: Solid Earth*, *89*(B6), 4298-4312.

Kronenberg, A. K., & Tullis, J. (1984). Flow strengths of quartz aggregates: grain size and pressure effects due to hydrolytic weakening. *Journal of Geophysical Research: Solid Earth*, *89*(B6), 4281-4297.

Rybacki, E., & Dresen, G. (2004). Deformation mechanism maps for feldspar rocks. *Tectonophysics*, *382*(3), 173-187.

Violay, M., Gibert, B., Mainprice, D., Evans, B., Dautria, J. M., Azais, P., & Pezard, P. (2012). An experimental study of the brittle, ductile transition of basalt at oceanic crust pressure and temperature conditions. *Journal of Geophysical Research: Solid Earth,* 117(B3).
